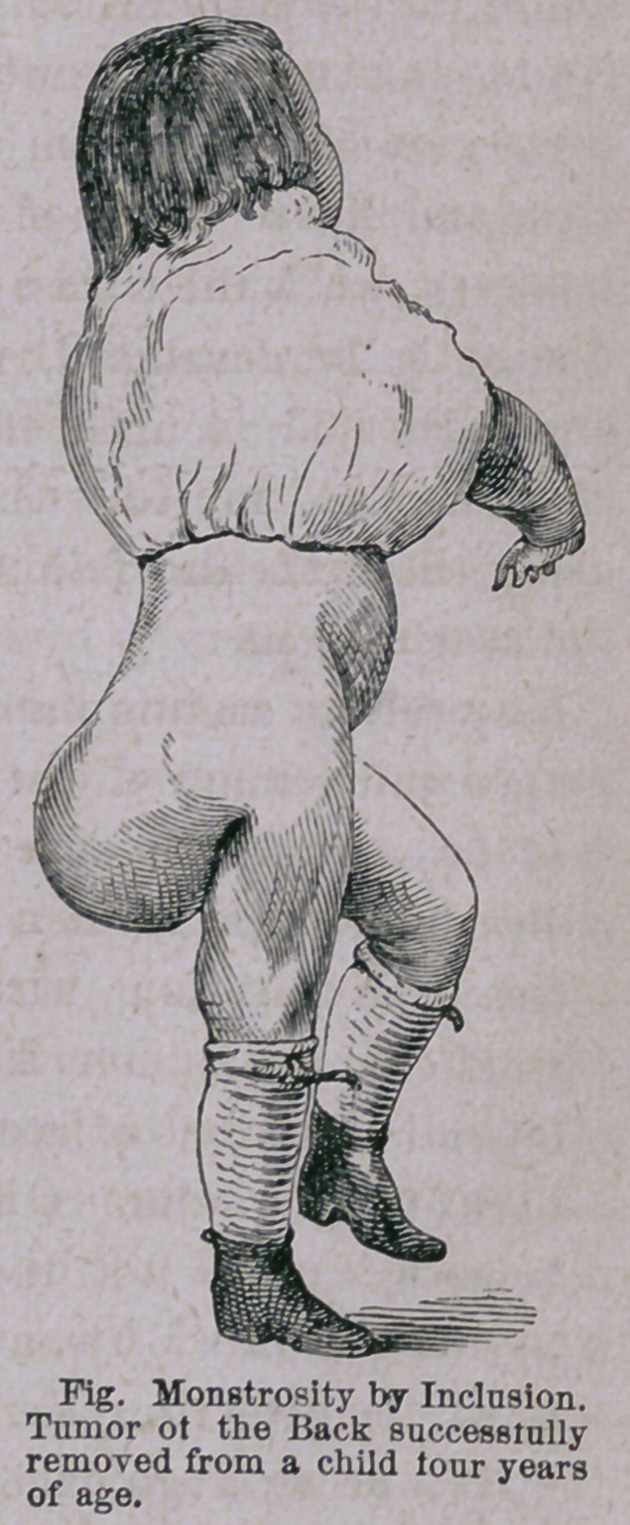# Clinical Remarks upon Surgical Cases

**Published:** 1875-04

**Authors:** Julius F. Miner, W. W. Miner


BUFFALO

### Medical and Surgical Journal.

VOL XIV. APRIL, 1875. No. 9.

### Original Communications.

ART. I.—*Clinical Remarks upon Surgical Cases occurring in the Buffalo Hospital of the Sisters of Charity. By Prof. Julius F. Miner, M. D.* Reported by W. W. MINER, M. D.

CASE XVII.—*Monstrosity by Inclusion. Successful Excision.* Myrtie M.——, four years of age, living in Genesee county, has since her birth, had in the region of her sacrum and nates, a large pendulous tumour, which entirely obliterates the cleft naturally separating the nates, and appears as a single protuberance of large size, depending from a firm attachment to the spinal column. It is about ten inches in diameter laterally, flattened from before backwards, and measuring in an antero-posterior direction about eight inches, while it depends several inches below the level of the perineum. Notwithstanding its great size, the child is able to sit, and can run about, though there is a bending forwards of the body in standing or walking, compensatory for the extra weight carried behind. At birth, the tumor was about the size of a pint measure, it was then punctured, at the earnest solicitation of friends, and about one teacupful of clear serum flowed; collapse of the cyst following. The point of puncture has never entirely closed, and the secretion which still escapes from it, is getting to be objectionable by its presence and bad odor. The tumor has increased in size relatively with the growth of the child, though with the advance in years, the inconvenience of the growth is attaining greater importance. The parents are anxious to have the deformity removed if possible, and are willing to risk considerable in order to rid their child of this obnoxious and harassing appendage.

The case is a rare one, and in examining it at my office, just before coming here, it presented quite a doubtful character. My first impression was that it was a hydro-rachitic cyst, having had at one time, if not at present, connection with the spinal canal through the aperture of a bifid spine. The fact that the child is now in perfect health, was not disturbed by evacuation of the fluid contents of the cyst at birth, and is not now affected by manipulation and careless treatment of the protuberance, seemed to cast doubt on the supposition of its having connection with the spinal cord. It is regular in outline, distinctly circumscribed, except in the region of the sacrum and coccyx, where its attachment seems close and firm. It is of firm consistence, certain portions of it, however, feel harder than others, as if it were multilocular or indistinctly lobulated. Drs. Rochester, Boardman, and others who are interested in examining it, while regarding it of doubtful character, do not feel that the indications are such as to forbid using the knife and finding out what the practicable treatment of the case may be.

Chloroform having been administered, a cautious dissection and partial enucleation of the growth from the overlying integument is made; the line of the two incisions being curved outwards on either side of the median line enclosing an oval piece of integument. Separation without appreciable hemorrhage was thus effected of the tumor from its surroundings laterally, but firm attachment existed adjacent to the sacrum, also to the rectum, in all four or five inches in length, and an inch and a half in breadth. The coccyx could not be felt or found. The walls of the rectum were not separated, by any appreciable interval, from the parietal wall of the growth. Very careful dissection, with the finger in the rectum as a guide, effected at length, separation here. The principal and supporting part of the pedicle was of doubtful character, appearing very firm, fibrous or fibro-areolar in structure. Incision of this was finally made, when it appeared that there was no canal existing between the growth and the spinal cord. The vascular connections were not of great size, two ligatures only being used.

It was then found that there was an entire absence of the coccyx in the child. The sacrum also was greatly deficient, though it was such as served to complete the pelvic arch and furnish a basis of support and connection with the spine. The operation occupied considerable time, and made quite a demand upon the little patient's powers temporarily.

The accompanying cut, photographed on wood and engraved, is a faithful representation of the external appearance of the appendage before removal.

Fig. Monstrosity by Inclusion. Tumor of the Back successfully removed from a child four years of age.

The weight of the tumor removed was four and one half pounds. It was of spheroidal form, cystic in external appearance. Examination of the growth, with the aid of a knife, was made. It was found that the growth had been enclosed in the integument of the back and nates much as the ovum is enclosed in the uterus. Its external covering was more or less closely attached to the integument of the child, still had been separated from it by enucleation and dissection. Beneath this outer covering of the tumor, there was a space corresponding to an amniotic cavity, and in which, there was a cheesy substance resembling the vernix caseosa. On the surface of the enclosed part, were two protuberances, one-fourth of an inch in diameter, and suggesting by their appearance and relative position, the idea of two nipples. Further incision into the body of the growth, revealed its generally adipose and fibro-cellular character; one portion of about three ounces weight, was of dark color, gelatinous consistence, and had some resemblance to liver structure or to a softened coagulum of blood. An ounce or two of clay-colored and firm substance was considered to be meconium. In the harder, nodular portions of the growth, were found the last phalanx of a finger, perfect in its exterior of nail and integument, and with rudimentary phalanx of bone; also the articulated metacarpal and phalangeal bones of another finger, without proper integument. Further dissection revealed one of the pelvic bones, the left innominatum, and articulated with it, the upper third of the corresponding femur. Likewise, also, there were the articulated bones of a right leg and foot, with half the corresponding femur: the two former were greatly distorted, turned outwards as in club-foot. The bones of the fifth toe were absent, the others, however, were complete and furnished with nails and phalangeal integument. The innominate bone was of the size of that in the fœtus at the fourth month, while the tibia and fibula, and bones of the foot, which were most fully developed, were those of near the full term of fœtal growth. The length of the tibia is four and one-half inches.

The child rallied without delay, and completely, so that it appeared on the day following the operation to have been very little disturbed generally by it. On the fourth day of her stay in the hospital, an eruption appeared on the child which presented the appearance markedly of scarlet fever. She had a mild condition of fever the fifth day, which perceptibly declined the next day. Beside desquamation no other symptoms of scarlatina were manifested, so that its mildness caused doubt in some as to its scarlatinal character. The silk sutures were removed one week after the operation, union by first intention having occurred in the greater part of the wound. On the tenth day the child was taken home, being as near well as the time would allow, was able to stand on her feet, walk a little, and was feeling in great joy at sight of its father and the prospect of going home.

The growth was excised on the seventeenth of February. We learn from the parents that at the middle of March the child was running about, its recovery being well established.

Case XVIII.— *Vesical Calculus and Operation.*—Samuel R——, aged twelve years, has since infancy suffered from urinary disorder.

This has become so serious as to forbid his longer continuance at school, where enjoying special indulgences, he has until late been busied. He has incontinence of urine in notable degree, tenderness and pain in the hypogastric region, also that peculiar feeling of irritation in the extremity of the penis, which is characteristic of vesical calculus. His general health, however, seems not impaired, in fact, he is in a very good condition of flesh and vigor. He has just come in here, and we will examine him with a sound, to find if possible the cause of his troubles.

Partial anæsthesia was induced and examination made by experienced observers, but no evidence of calculus obtained at this time. The boy was given anodyne and diuretic medicine which afforded him much relief: meanwhile his urine was examined, and found in no way abnormal, except it contained mucus to a notable extent, a few blood corpuscles and abundant phosphates. A silver catheter was used when difficulties were particularly troublesome, and in this way impact with the calculus was rather accidentally obtained. The impact obtainable was of but the slightest degree of force, though positive in character. It was obtainable only in a very limited area in front and above the vesical neck.

The operation for removal of the calculus was performed on the twenty-third of February, in presence of the students, the Curators of the College, many of the Alumni and others who were in attendence at the exercises of Commencement. The remarks were much as follows:

Those of you who make examination of this boy, will agree with me doubtless as to the positive indication now obtained of the presence of stone in the bladder. Only a small amount of water can be injected into the bladder, and this causes such powerful contraction that we gain no information by this means. The calculus seems to be located in the anterior part of the bladder, and is more or less fixed in its position, so that no proper idea of its size can be formed. It seems as if it were nearly encysted in the walls of the bladder, and may not properly be subject to removal. Still the fellow is altogether disabled by his trouble, which unrelieved must result disastrously, and as I have had most happy results in the dilatation of the the neck of the bladder, I do not think in this case, that satisfactory exploration of the bladder with the finger, is unwarranted, even though removal of the calculus may not be accomplished.

The method of operation proposed, is opening down upon the membranous portion of the urethra, and the making in it of a slit suitable for the introduction of a probe into the bladder by the side of the director, with which instruments and the finger, dilatation of the neck of the bladder is then to be undertaken, without making any farther incision than that just mentioned. In this way the neck of the bladder in a young patient may be made to dilate to a remarkable degree. A grooved staff having been introduced, incision was made into the membranous portion of the urethra. The perineal arteries flowed freely when severed, but soon ceased this of themselves. Having introduced a probe by the side of the director, I can as you notice, gradually enter my finger as a wedge between these instruments into the bladder, and am now able to sweep it around to some extent within. Sufficient dilation has been made to allow the introduction of forceps suitable for seizure of the stone.

With cautious manipulation, it was found that the blades of the forceps, having been opened in contact with the stone, could be sufficiently insinuated between the closely encircling walls and the sides of the stone, as to allow dilatation of the capsular constriction by further opening of the forceps, and at length, the proper seizure of the stone, which was then found to be of great size.

By a gradual process of dilatation and delivery as in instrumental obstetrics, the canal of egress was made to allow the passage of the calculus as held in the grasp of the lithotomy forceps. Several minutes were occupied in the delivery of the stone, and though a great amount of dilatation was necessary, still only very cautious efforts of extraction were made. Happy delivery of the stone was at length effected, when it was found to have been very favorably grasped in its transverse diameter.

The calculus measures two and one-eighth inches in length, one and one-half in breadth, and one and one-eighth in thickness, and is of a flattened ovoidal shape. Prof. Geo. Hadley accurately reports the weight of it to be seven hundred and six grains or 45.75 grammes. Its specific gravity has been found to be nearly

1.55. It is of a moderately smooth exterior and presents many characteristics of a uric acid calculus, which is generally looked for in persons thus young in years.

The boy was in no way whatever disturbed by the operation. He remained in bed until his urine was discharged naturally, and the perineal wound closed. At first he had trouble in controlling his bladder; gradually gained power over the sphincter vesicæ, so that by careful attention to natural requirements he was able to retain and expel his urine in a complete manner. He left the Hospital on the last of March.

This case is a very remarkable one: very rarely at least is it paralleled. That recovery not alone, but absence of all general symptoms of after disturbance, should result after removal of a vesical calculus of the aforesaid dimensions, where dilatation of the neck of the bladder was made, sufficient to allow the passage of a stone and forceps measuring four and one-half inches in circumference, would seem scarcely possible to those who have not had personal observation of the same. Dolbeau, after numerous and careful experiments, has placed the limit of practicable dilatation of the prostatic urethra at thirteen sixteenths of an inch. Besides rupture, authors regard paralysis as forbidding dilatation beyond these narrow limits.

Since the above was in print, on the 24th of April, we found the boy at work in a brewery, and learn from himself and friends that he has complete control of his bladder and has such freedom from troubles as he has never before known. He has not wet his bed for two weeks, seldom rises during the night, and not more than once, to pass water; urine clear; a slight feeling of irritation sometimes felt in the bladder just after its evacuation seems the only indication left of his former troubles.

## Figures and Tables

**Fig. f1:**